# Patient and interest organizations’ views on personalized medicine: a qualitative study

**DOI:** 10.1186/s12910-016-0111-7

**Published:** 2016-05-13

**Authors:** Isabelle Budin-Ljøsne, Jennifer R. Harris

**Affiliations:** Centre for Medical Ethics, Institute of Health and Society, University of Oslo, P.O Box 1130, Blindern NO-0318 Oslo, Norway; Norwegian Cancer Genomics Consortium, kreftgenomikk.no, Oslo, Norway; Department of Genetics and Bioinformatics, Norwegian Institute of Public Health, Oslo, Norway

**Keywords:** Personalized medicine, Patient organizations, Genetics, Ethics, Precision medicine

## Abstract

**Background:**

Personalized medicine (PM) aims to tailor disease prevention, diagnosis, and treatment to individuals on the basis of their genes, lifestyle and environments. Patient and interest organizations (PIOs) may potentially play an important role in the realization of PM. This paper investigates the views and perspectives on PM of a variety of PIOs.

**Methods:**

Semi-structured telephone interviews were conducted among leading representatives of 13 PIOs located in Europe and North-America. The data collected were analysed using a conventional content analysis approach.

**Results:**

The PIO representatives supported the realization of PM but feared that many financial, structural and organizational challenges may delay its realization. They encouraged strategies to modernize drug licencing mechanisms, develop research and data sharing infrastructures, and educate patients and health care professionals in PM. Notably, they emphasized the importance of developing PM in an equitable way and taking into consideration the patients’ needs, values and personal situation. Despite varying levels of awareness regarding PM, the PIO representatives expressed willingness to engage in the PM agenda and recommended that PIOs work closely with policy-makers to design PM in a way that truly addresses the needs and concerns of patients.

**Conclusions:**

PIOs have the potential to become central drivers of the PM agenda. Collaborations should be further developed between PIOs, researchers, drug developers and health care authorities.

## Background

Recent advances in biomedical research and biotechnology offer new possibilities to tailor prevention, diagnostic and treatment to the specific needs of patients. By utilizing information about the patients’ genetic and molecular profile combined with their family history and clinical and environmental data, it is hoped that health care providers will be able to start interventions earlier and select therapies that are more precise, efficient and provide less side-effects – strategies broadly known as personalized medicine (PM) [1]. Large investments are being made worldwide to support such strategies. In the United States, the $215 million Precision Medicine Initiative [2] was recently launched with the objective to accelerate biomedical discoveries and improve the effectiveness of treatment. In Europe, the European Commission has since 2007 committed over €1 billion of health research funding to the development of ‘-omics’ technologies and targeted therapies, and more funds are expected to be released in the coming years [3]. PM has been extensively described in recent reports from national and international medical organizations and funders [4–6]. These reports emphasise the importance of engaging a variety of stakeholders such as health care providers, biomedical researchers, regulators, drug developers, patients and patient organizations in driving PM forward. Among these stakeholders, patient organizations are particularly valuable players regarding their role in patient education [7], assessing new biomedical developments [8], and supporting research projects [4]. However, with the exception of recent surveys in which patient organizations identified potential barriers in PM implementation [9] and key priority areas within cancer [10], little empirical data are available to document the views and perspectives of patient organizations on PM.

Investigating how patient organizations perceive PM and which role they are willing to play in its realization is important for several reasons. First, patient organizations have close contact with the communities of patients they represent. They are therefore well-placed to provide qualified opinions regarding how to implement PM in a way that addresses the needs and concerns of these communities. For instance, PM may require that patients learn about their genetic risk profile, engage more actively in the medical decision-making process and share their health data with researchers and clinicians more broadly than conventionally practised [11]. Patient organizations are well-qualified to familiarize patients and communities with such developments. Second, patient organizations may provide useful guidance in addressing potential ethical and societal challenges that may arise when new medical approaches are implemented. For instance, PM may imply that patients are offered differential access to treatment depending on their genetic profile [12]. Patient organizations may be well placed to explore how dialogue with patients and communities should take place when access to conventional treatment may be restricted. Third, patient organizations have comprehensive expertise within the areas of patient and health care professional capacity building [13, 14], the development of online patient communities [15], the design of regulatory frameworks and patenting policies [16], the financing and management of clinical trials and biobanks [15, 17–19], and more recently, in developing databases and web-based platforms for patient-driven data sharing [13, 20]. Making use of these skills and experiences may accelerate the realization of PM.

Although patient organizations are often referred to as one homogenous group, they are rather a constellation of entities that vary considerably in size, organizational structure, denomination and mandate [13]. For instance, patient organizations may be publicly or privately-funded, small entities which represent only a limited network of families suffering from a particular condition, or they may be large-scale, international organizations bringing together national organizations which focus on one specific disease or specialize in improving the living conditions of all patients. We conducted a qualitative interview study among leading representatives of non-profit patient organizations which are concerned with one specific disease or disease area and define themselves as patient advocacy organizations, umbrella organizations for patient advocacy organizations or interest organizations, hereafter referred to as patient and interest organizations (PIOs). The objective of our study was to collect information about three main issues: 1) the PIOs’ views and perspectives on PM, 2) recommendations for facilitating the realization of PM, and 3) the role they foresee that they may play in the realization of PM.

## Methods

### Recruitment of study sample

To collect information about the views of a variety of PIOs, we proceeded in two steps. First, in July 2014, we sent an e-mail invitation to the leaders of a small number of PIOs that co-operate with the research network of the Norwegian Cancer Genomics Consortium (NCGC) [21], a national research platform aiming to establish personalized clinical strategies for cancer treatment in Norway. The email invitation provided an outline of the study objectives and a description of what participation in the study entailed. In the invitation, PM was described as “an emerging practice of medicine that uses an individual’s genetic profile to guide decisions made with regard to the prevention, diagnosis, and treatment of disease” as defined in the Talking Glossary of Genetic Terms of the National Human Genome Research Institute [22]. The email invitation included a request for written informed consent. The study was approved by the Norwegian Social Science Data Services.

The first PIO leaders who accepted our invitation helped us identify other PIO representatives that might want to participate in the study. Through such snowball sampling, we were able to recruit representatives from 8 PIOs concerned with one specific disease or disease area (6 PIOs in Norway, 1 in the United Kingdom and 1 in the USA) between July 2014 and January 2015. In parallel and in order to extend our sampling, we sent email invitations to 20 leaders of disease-specific PIOs members of the European Patients’ Forum (EPF) [23], an umbrella organisation of pan-European patient advocacy organizations. The PIO representatives were recruited in the study until a point of saturation was attained, i.e. when no significant new information was collected. Five PIO representatives agreed to join the study, six responded that they did not have time to participate or did not want to participate, and nine did not respond to our invitation. In total, thirteen PIOs working within the areas of cancer (4), hereditary and genetic disorders (3), mental health (1), diabetes (1), psoriasis (1), AIDS (1), lupus (1), and primary immunodeficiencies (1) participated in the study (Table 1). The representatives interviewed were organizational leaders (10) (e.g. CEO, secretary general, director) and senior managers (3) employed by the PIOs. As leading representatives of their PIOs, they were able to speak on behalf of their organization although many specified that their organization did not have an official position on PM. The size of the PIOs varied from small PIOS gathering families suffering from a rare genetic disorder to large PIOs gathering more than a thousand organizational members.

**Table 1:** PIOs participating in the study

PIO name	Membership	Country
DEBRA Hrvatska (Dystrophic Epidermolysis Bullosa Research Association)	Approx. 50 families	Croatia
Genetic Alliance UK	>100 organizational members	UK
Genetic Alliance USA	>1,000 organizational members	USA
LUPUS Europe	Approx. 22 country memberships	Denmark
Sarcoma	Approx. 350 individual members	Norway
The European AIDS Treatment Group	Approx. 110 individual members	Belgium
The European Umbrella Organisation for Psoriasis Movements (EUROPSO)	Approx. 20 country memberships	Norway
The International Patient Organisation for Primary Immunodeficiencies (IPOPI)	Approx. 55 country memberships	UK
The Norwegian Breast Cancer Society	Approx. 14,500 individual members	Norway
The Norwegian Cancer Society	Approx. 113,000 individual members	Norway
The Norwegian Childhood Cancer Organization	>3,000 individual members	Norway
The Norwegian Council for Mental Health	Approx. 29 organizational members	Norway
The Norwegian Diabetes association	Approx. 40,000 individual members	Norway

### Data collection and analysis

Semi-structured telephone interviews were conducted with the thirteen PIO representatives, lasting forty minutes on average. An interview guide was used to lead the conversation, which included open-ended questions about the PIOs’ perspectives regarding PM, PM-related activities, perceived challenges with regard to the realization of PM, recommendations for the adoption of PM, and the potential roles the PIOs may play in the realization of PM. The interviews were conducted in English or Norwegian by the first author trained in qualitative methods and fluent in both languages. The interviews were audio recorded and transcribed verbatim in English and Norwegian. The transcripts were analysed manually by the first author using a qualitative content analysis approach in an inductive way [24]. First, a thematic analysis of the interview texts was conducted to identify overarching themes that emerged from the responses to each open-ended question formulated in the interview guide. Next, the substantive content of the text was extracted, coded and categorized according to the overarching themes. If new codes were identified, the coding frame was updated accordingly. Then, the text pertaining to each of these themes was condensed to reflect the main points raised by the PIO representatives. In August 2015, a two-page report summarizing the main findings was sent by email to the PIOs representatives for validation. Their comments and corrections to the report were integrated in the results.

## Results

The PIO representatives described their interest in PM and potential challenges that may impede or delay the realization of PM. Particular emphasis was put on possible side effects of focusing on PM strategies. Then, the representatives made a number of recommendations for the realization of PM and discussed how their organizations may contribute to the PM endeavour.

### PIOs interest in PM

Overall, the PIO representatives expressed interest in PM. They explained that the current medical needs of their patient groups are largely unmet and medical strategies which address issues of side effects, overtreatment and undertreatment are strongly needed. As outlined by this representative:

(1) If you create a medicine that directly targets the individual, you will then be able to avoid all these unsuccessful attempts to find the right treatment without getting better, having to deal with the side effects, the downturns, the disappointments (…) when you use a medicine that has side effects for a long period, you are sick for months and then it turns out that [the medicine] does not help anyway. Developing medicines that will perhaps work at once and not after the fifth attempt, (…) we see this as perhaps the most important thing to achieve.

However, several representatives confessed that PM was a topic that had not been thoroughly discussed in their organization. Most PIOs did not use the terms PM and did not specifically mention PM in their strategic documents with the exception of some cancer PIOs. Several representatives also explained that the concept of PM and its possible practical implications were still unclear. As an illustration, one representative believed that PM meant that health care professionals allocate more face-to-face time with each patient.

### PM challenges

#### High cost of PM

Most representatives feared that PM may be too expensive for many health care systems which are currently dealing with significant financial constraints. They experienced that patient access to conventional treatment is increasingly restrained due to cost issues, and observed that new targeted drugs that are launched on the market are so highly-priced that patients can hardly afford them unless their cost is fully covered by payers. As expressed by this representative:

(2) If we end up with the same level of cost to get an authorization for a targeted drug that would effectively treat 10% of a population of people with a given disease, rather than being used to treat a 100% of the population with a given disease, then the cost for (…) personalized medicine per patient treated will have to be higher.

Although the representatives acknowledged PM’s potential to save costs as treatment becomes more targeted and waste is avoided, they expected PM to require significant up-front capital investments in equipment and infrastructures that most countries cannot afford. They also suspected that PM may lead to increased medical follow-up as genetic tests are more frequently used in clinical settings to confirm a diagnostic. Another concern was that patients who purchase genetic testing kits from direct-to-consumer genetic testing companies may seek medical advice from their physician to interpret the results, thus creating additional burdens on health care services.

#### Lack of health literacy

Several PIO representatives expressed scepticism regarding the patients’ ability to understand and endorse PM strategies. They noted that patients often struggle to understand basic medical information:

(3) People do not understand information well enough, this is where we see a big job for the patient organizations and patient groups in each of the countries in Europe, (…) educate, bring awareness of how important (…) the different kinds of medications are, when they are supposed to be used and for what, (…) there is a reason why it has been prescribed (…). There is a huge communication aspect that we have just picked up.

Another representative suspected that many patients are not ready to learn about their genetic profile, in particular if unexpected findings are discovered:

(4) Genetic testing for rare genetic conditions and other disorders can now be performed in hospitals, you can get some answers that are incidental and that no one asked for and for which a decision must be made. We think that this is a challenge (…), the patients are not aware of this. They can get a lot of information that is hard to digest.

These views were however nuanced by the perspectives of other representatives who emphasized that patients who have been waiting for a diagnostic for many years are often eager to learn about their genetic profile. Several PIO representatives also explained that general practitioners often do not understand the specificities of disease, and suspected that many are insufficiently trained in genetics to use PM strategies in their medical practice. Similarly, several representatives explained that policy-makers often do not understand why patients need specific types of treatment rather than a “one-size-fits-all” treatment.

#### Lack of mechanisms and infrastructures to support PM

Several PIO representatives explained that current drug licensing mechanisms unnecessarily delay the launching of personalized treatments. Receiving drug approval from regulatory authorities often takes years and is particularly cumbersome if such approval is needed across borders. Although the representatives largely believed that data protection regulations are needed to protect the rights and interests of patients, some representatives suspected that such regulations may be too stringent and hinder the conduct of biomedical research. As expressed by this representative:

(5) When it comes to access to this information in relation to biobanks, there are so many rules already dealing with this, I know that many are concerned about this (…), but what is it we are scared about? There are rules for how [information] can be managed and used, I do not see any danger that [information] will be misused.

#### Unwanted consequences of PM

In general, the PIO representatives worried that PM, because of its high costs, may reinforce already existing health care disparities among social groups unless specific measures are taken by policymakers to enable equitable access to PM. Some representatives also expressed concerns that patient groups may be forgotten if they represent a genetic subset that is not considered lucrative by pharmaceutical companies. As expressed by one representative:

(6) If it is really individualized, if it’s really about genetics, there will be populations that will be marketwise less interesting, maybe population-wise they are, meaning that you should not only look at the benefit for the rich white community but also for the populations that are maybe research-wise less interesting to include but might really benefit from more research.

Some representatives feared that the increased use of technology in health care may lead professionals to reduce patients to their genetic profile. As described by this representative:

(7) I think it is essential that the patient remains at the center and that he doesn’t become a data or an entity; it’s really a patient and a face and the [health care professionals] need to treat that patient, that’s the main goal.

Genetic discrimination was a shared concern of some representatives. An example was provided of patients being denied health insurance because of their medical history even if the law prohibits such practice. As expressed by one representative:

(8) If we, sometime in the future, find genes that you would like to have and genes you would prefer not having, it is clear that it may contribute to creating A-people and B-people. From the moment our genes become a question of value, it may create differences between people. Some people may be worth more than others and then we are back to the 30’s, so from a historical point of view, this is important.

Finally, one representative worried that priority may be given to genetic research, not because it may lead to the most useful results, but because it is technologically exciting and may benefit the commercial interests of pharmaceutical companies. This representative emphasized the importance of also conducting other types of research such as social and behavioural research.

### Main recommendations for the realization of PM

The PIO representatives made recommendations for the realization of PM that can be assigned to five main categories as described below and summarized in Table 2.

**Table 2:** PIOs’ recommendations for the realization of PM

Recommendations
Policy-making
• Establish principles and criteria for equitable patient access to PM
• Modernize drug licensing mechanisms, e.g. through adaptive pathways
• Allocate specific funding to the implementation of PM
• Implement PM gradually, e.g. through pilot projects including the most needy patients
Patient-centered health care
• Provide simple, actionable genetic information to patients; ally with genetic counselors
• Take into consideration the patients’ values, personal situation and health literacy level
• Protect the patient’s right not to know
• Educate patients and health care professionals in PM strategies
Increased, inclusive research
• Conduct more basic/epidemiological research
• Invite patients and PIOs early in the planning and design of clinical trials
• Broaden eligibility criteria to recruit more patients in research
Data sharing and protecting privacy
• Develop privacy-solid biobanks and data sharing infrastructures
• Ask for permission before using personal health data
• Develop flexible and interactive consent mechanisms
PIOs’ active participation in agenda setting
• Engage as early as possible in the development of PM
• Contribute to educate stakeholders in PM
• Contribute to design the PM agenda, e.g. by identifying priority areas
• Support the development of collaborative research projects
• Respond to national hearings of relevance for PM
• Join research ethics committees and drug approval boards
• Develop partnerships with medical professions, e.g. through medical expert panels
• Develop partnerships between PIOs nationally and internationally
• Develop partnerships between PIOs and researchers, medical professions, and policy makers

#### Policy-making

Overall, the representatives emphasized the importance of providing equitable access to PM. They explained that access to genome sequencing technologies and targeted drugs should not be limited by financial constraints but offered according to specific priority criteria that are jointly established by policymakers, health care professionals and patient representatives. They believed that modernizing drug licensing mechanisms is necessary to enable quicker access to targeted drugs. Adaptive pathways mechanisms were mentioned as a potential approach to improve timely access for patients to new medicines [25]. The representatives also recommended allocating specific funding to the implementation of PM, for instance to develop necessary biobanks and data sharing infrastructures. Some representatives suggested implementing PM gradually, starting with the patients groups that are most in need such as those for whom no established treatment currently exists.

#### Patient-centered health care

Most representatives emphasized the importance of an open dialogue between health care professionals and patients. They believed that patients should be provided with information that is simple, understandable, actionable and adapted to the patient’s needs, values, level of health literacy and way of dealing with disease. They explained that the value of an intervention should not only be measured in medical, biological or scientific terms but also in terms of how it impacts the patients’ quality of life and ability to function in society, and recommended that these aspects are taken into consideration when assessing patient needs. They strongly recommended providing genetic counselling to patients when genetic information is available. In general, the representatives believed that the medical literacy of patients should be improved. However, they explained that patients differ in their willingness to learn about their disease and engage in their health care, and that their personal preferences, for instance regarding access to their genetic information, should be respected. In addition, the representatives considered it important to educate health care professionals in PM strategies. In contrast, educating the general public about PM was seen as useful but not an absolute priority. The representatives believed that public awareness about PM may gradually develop as targeted treatments are becoming more broadly available in health care.

#### Increased, inclusive research

In general, the representatives believed that more research should be conducted to understand the causes underlying disease, investigate the mechanisms of side effects, explore the consequences of living a long time with a specific treatment and develop strategies to improve the quality of life of patients. They explained that patients are willing to participate in medical research but are often denied such opportunity because of strict eligibility criteria. They recommended engaging patients and patient representatives as early as possible in the planning and design of clinical trials and broadening eligibility criteria to include more varied populations across socio-economic groups.

#### Data sharing and protecting privacy

The representatives supported the development of biobanks and data sharing infrastructures that are governed by solid and transparent privacy protection frameworks as these are essential to maintain the trust of patients and protect their interests. Privacy protection was seen as particularly important when the health data that are shared originate from patients suffering from socially stigmatizing diseases. The representatives explained that more work is needed to make people comfortable with sharing their personal health data.

#### PIOs’ active participation in agenda setting

The representatives believed that PIOs should be involved as early as possible in the planning and design of the PM agenda. They explained that the main role of PIOs in PM includes helping to: a) increase literacy in PM among patients and health care professionals, b) identify the areas in which PM is most needed, and c) support the development of research projects that are attractive to patients. The representatives envisioned increased collaboration between PIOs and research groups, medical bodies, drug developers and policy-makers, nationally and internationally to encourage the development of PM. They also believed that patients and lay representatives should be more frequently invited to join research ethics committees and drug approval boards. The representatives recommended the development of inter-PIOs collaborations to enable the organizations which are more knowledgeable about PM to help others join the PM endeavour. Finally, they called for initiatives to educate PIOs in PM as finding the right information about PM may be challenging and many PIOs do not have a clear understanding of how they can contribute to the successful realization of PM.

## Discussion

Our findings show that PIO representatives had a positive although cautious attitude toward PM. They believed that PM is needed but suspected that many financial, structural and organizational challenges may delay its realization. Although the recommendations forwarded by the representatives to support the realization of PM are largely congruent with those made by the promoters of PM [4–6], they also shed light on specific ethical and societal challenges that may arise in the process of adopting PM. Importantly, attention to these considerations has not been emphasized in the public debate but they should be addressed to enable the successful realization of PM in a way that truly addresses the needs and concerns of patients.

Developing PM in an equitable way, and involving discussions with decision-makers about how such equity may be achieved, was seen as the main priority. The PIO representatives based their rationale on the types of problems that their patients encounter daily in health care systems. For instance, the prices of new targeted drugs have steadily increased during the last decade [26] and health care systems frequently deny patients access to such drugs because of financial constraints unless the patients can pay out-of-pocket [27, 28]. The PIO representatives expressed concern that issues of social injustice may arise if individuals who are more socio-economically advantaged benefit most from new treatments because they have the financial ability to pay for them. The fundamental principle of equitable access to health care may be threatened when “niche buster drugs” designed for smaller groups of patients are more expensive and therefore less accessible than blockbuster drugs [28]. This issue has already been raised by coalitions of patient organizations which have called for an equitable and universal patient access to PM independent of the patient’s socio-economic status and geographical location [9, 29, 30].

Although the PIO representatives emphasized that equitable access to PM was critical, they did not articulate in detail what such equity should entail. Equity is a broadly acknowledged ethical concept that is concerned with the fair and impartial “distribution of benefits and costs to distinct individuals or groups” [31]. Although most health care systems strive to achieve equity, it has shown to be difficult to operationalize. For instance, it is usually considered as equitable to allocate health care resources on the basis of consideration of the actual needs of patients [32]. However, how to determine which needs are most important, and how they should be prioritized is often challenging. As an illustration, should the needs of terminally ill patients be addressed in priority, or those of patients who may yield significant gains in life expectancy if treatment is provided [33]? Two PIO representatives mentioned that patients for whom no standard treatment exists, for instance some groups of cancer patients, should be prioritized to receive access to genome sequencing in the hope that a potential treatment may be found. However, neither the importance nor the implications of providing genome sequencing to cancer patients if little is known about the genes involved in their cancer types are well understood. Other criteria may also have to be taken into consideration for resource allocation such as the efficacy and effectiveness of the intervention (its ability to lead to a positive outcome for the patient) and its cost-effectiveness (ability to provide value for money) [34]. Even if it is seen as morally justifiable to give priority to the patients who are in greatest need, it may not be wise from a cost-effectiveness perspective if the intervention is not expected to benefit the patient. Decision-makers are faced with having to balance between these different considerations (equity, efficacy, cost-effectiveness) and make decisions that do not unjustifiably discriminate some groups of patients. In the absence of a clear normative framework providing guidance on how to distribute resources fairly [35], it may be useful to investigate processes that are deliberative, transparent, and “appeal to rationales that all can accept as relevant to meeting health needs fairly” [36]. This is where PIOs may play an important role by collaborating with decision-makers in order to find pragmatic solutions for an equitable and reasonable resource allocation. As an illustration, the Scottish Medicines Consortium convenes clinicians, health economists and patient representatives to identify and prioritize those medicines and interventions which represent good value for money to patients and should be launched on the market [37]. Such collaboration may be good example of how a fair process may take place.

Taking into consideration patients’ interests and values to a greater extent than traditionally practised was also seen as critically important by the PIO representatives. PM is often described as a holistic approach to medicine that utilizes information about the patient’s clinical and family history, genetic susceptibility to disease, response to certain drugs, and lifestyle to assess the patient’s health [4–6]. The PIO representatives interpreted the meaning of “personalized” medicine slightly differently from the promoters of PM. Although the PIO representatives largely agreed that focusing on the biological and environmental factors that may affect the health of the patient is important, they also believed that “personalizing” health care means that greater attention should also include taking the time to listen to the patient, learn about her values, assess her mental and spiritual well-being, understand her personal circumstances, and improve her quality of life and ability to function in society with a condition. The views expressed by the PIO representatives are in concordance with recent calls for a more “humanistic” medicine that approaches the patient as a whole person with a personal history, emotions, beliefs and sufferings [38]. A humanistic doctor takes the time to listen to the patient’s story, shows compassion and empathy, makes sure that the patient feels valued and respected, and takes into consideration the patient’s personality and experience in the process of medical care. As the PIO representatives repeatedly emphasized during our interviews, no technological advancement or biological instrument can substitute the importance of the doctor-patient relationship.

PM aims to strengthen dialogue and collaboration between doctor and patient through increased patient engagement [8]. In accounts of PM, patients are encouraged to discuss various treatment options with their healthcare provider, communicate about their preferences, values and lifestyle, and provide feedback on health care processes and outcomes [8]. At such, patient engagement may be a critical strategy to enable the provision of health care that is responsive to the patient’s specific needs and situation as envisioned by the PIO representatives. Patient engagement enables a better power balance between patients and doctors, thus making it easier for patients to express their concerns and preferences, and bring perspectives on their own care [8]. However, while patient engagement strategies may work for those patients who are eager to take responsibility for their health care, seek knowledge and understand the details of their clinical situation, they may not be suitable for those who prefer to delegate the majority of the responsibility for their health care to their health care professional [8]. Similarly, some patients may not have the ability to engage in their health, for instance because they lack the necessary health literacy to understand what is at stake [39]. In this context, the PIOs that have available resources and are willing to engage in PM may have an important role to play in enhancing the health literacy of patients and familiarizing them with engagement strategies. Other patients may be so physically and mentally ill that they do not have the capability to engage. For these patients, providing health care with compassion and empathy may be particularly important; time should be allocated in the PM agenda to enable health care professionals to interact in a “humanistic” manner with their patients.

Finally, our study results indicate that levels of awareness regarding PM vary considerably between PIO representatives. In general, the representatives from the larger PIOs were more familiar with the concept of PM and worked more mindfully toward PM than representatives from smaller PIOs. Similarly, cancer PIOs were more likely to integrate the concept or aspects of PM in their strategy independent of their organizational size. Larger PIOs may have more resources to investigate new medical strategies than smaller PIOs which struggle with basic issues such as guiding their patients through the health care bureaucracy or educating health care professionals. Engaging in PM may be more challenging when the organizations’ resources are limited. Similarly, the general attention given to cancer in the PM discourse and the fact that most targeted drugs that have been marketed are cancer drugs [40] may explain why cancer PIOs were generally more aware of PM than non-cancer PIOs. As an illustration, several representatives from non-cancer PIOs believed PM to be primarily focusing on cancer to the disservice of other disease areas; a belief previously observed among health care professionals [41] and patient representatives [9]. It is therefore important that decision-makers work to increase levels of awareness regarding PM among PIOs, and provide support to those PIOs that have limited resources and competency but are willing to contribute to the PM agenda.

## Conclusions

The development and realization of PM requires the involvement of PIOs. Historically, PIOs have shown an impressive ability to engender important changes on a large scale that benefit patients. If the PIOs become sufficiently convinced that PM is the future of medicine and will benefit their patients, then they have the potential to play a significant role in driving the PM agenda forward. It is therefore important that researchers, health care funders, drug developers and policy makers invite PIOs to the table, not only the biggest and most influential PIOs but also the smaller ones should be engaged so that PM is developed in ways that address the “health care needs of patients. It is also important that PIOs work together to become effective advocates of PM. The European Patients’ Academy (EUPATI), a pan-European platform for patients and PIO engagement, may be a potential springboard for intra-PIO collaborations [42]. The European Alliance for Personalised Medicine (EAPM) [43], a coalition bringing together European healthcare experts and patient advocates, may also be one of the central arenas where PIOs could discuss with key stakeholders issues related to priority setting and resource allocation. Such models of collaboration and engagement are needed to give PIOs an increased opportunity to contribute to the decision making process regarding the design of PM.

### Study limitations

The results from our study provide insights into how PIOs working within a range of diseases perceive PM and reflect upon the PM agenda. Deciding on which PIOs to include in our study was challenging: there are thousands of active PIOs and they vary widely in organizational structure, areas of activity and funding. It was difficult to learn about the PIOs organizational and financial structure on the basis of the information they provide on their web site. In the absence of obvious criteria to select PIOs, we decided to restrict our sampling to non-profit PIOs that were disease-specific and were either member of the European Patients’ Forum, or were within the network of the Norwegian Cancer Genomics Consortium. This was a pragmatic choice that enabled us to come in contact with a variety of PIOs. However, our sampling may not be representative of the wider spectrum of PIOs that exist, and the PIOs situation (e.g. relationship to biopharmaceutical companies) could have to some extent influenced their interest in and level of knowledge about PM.

For many of the PIO representatives, this study was the first time they discussed PM and they were therefore not able to provide any concrete examples of issues related to their experience with PM, or specific recommendations regarding their potential role in the realization of PM. More work may be needed to investigate how a larger spectrum of PIOs which have specifically endorsed the concept of PM in their strategies, and work actively toward it, envision their role in the realization of PM agenda.

### Ethics approval and consent to participate

All participants in the study provided a written informed consent. The study was approved by the Norwegian Social Science Data Services (ref. 39155 / 3 / JSL).

### Availability of data and materials

The study data (interview records and transcripts) are stored securely at the University of Oslo and will not be shared according to requirements from the Norwegian Social Science Data Services, and conditions outlined in the informed consent.

## Abbreviations

PIO: patient and interest organizations
PM: personalized medicine
